# Clinical application of tumor volume in advanced nasopharyngeal carcinoma to predict outcome

**DOI:** 10.1186/1748-717X-5-20

**Published:** 2010-03-11

**Authors:** Ching-Chih Lee, Tze-Ta Huang, Moon-Sing Lee, Shih-Hsuan Hsiao, Hon-Yi Lin, Yu-Chieh Su, Feng-Chun Hsu, Shih-Kai Hung

**Affiliations:** 1 Department of Otolaryngology, Buddhist Dalin Tzu Chi General Hospital, Chiayi, Taiwan 62247; 2 Department of Oral and Maxillofacial Surgery, Buddhist Dalin Tzu Chi General Hospital, Chiayi, Taiwan 62247; 3 Department of Radiation Oncology, Buddhist Dalin Tzu Chi General Hospital, Chiayi, Taiwan 62247; 4 Department of Hematological Oncology, Buddhist Dalin Tzu Chi General Hospital, Chiayi, Taiwan 62247; 5 School of Medicine, Tzu Chi University, Hualian, Taiwan 97061

## Abstract

**Background:**

Current staging systems have limited ability to adjust optimal therapy in advanced nasopharyngeal carcinoma (NPC). This study aimed to delineate the correlation between tumor volume, treatment outcome and chemotherapy cycles in advanced NPC.

**Methods:**

A retrospective review of 110 patients with stage III-IV NPC was performed. All patients were treated first with neoadjuvant chemotherapy, then concurrent chemoradiation, and followed by adjuvant chemotherapy as being the definitive therapy. Gross tumor volume of primary tumor plus retropharyngeal nodes (GTVprn) was calculated to be an index of treatment outcome.

**Results:**

GTVprn had a close relationship with survival and recurrence in advanced NPC. Large GTVprn (≧13 ml) was associated with a significantly poorer local control, lower distant metastasis-free rate, and poorer survival. In patients with GTVprn ≧ 13 ml, overall survival was better after ≧4 cycles of chemotherapy than after less than 4 cycles.

**Conclusions:**

The incorporation of GTVprn can provide more information to adjust treatment strategy.

## Background

Nasopharyngeal carcinoma (NPC) is a unique malignant head and neck cancer with a specific behavior. It is rarely reported in the West but occurs at high frequency in Southern China, Hong Kong, Taiwan, Singapore, and Malaysia [1]. Radiotherapy has long been the standard treatment for patients with NPC because of its anatomic location and relative radiosensitivity. Based on the American Joint Committee on Cancer (AJCC) staging system in 1997, and the 5-year overall survival rates for tumor stages I, II, III, and IV were 95-70%, 83-65%, 76-54%, and 56-29%, respectively [2-6]. Although NPC is markedly radiosensitive, a high rate of treatment failure is observed in patients with advanced NPC especially distant failure. Combination chemotherapy plus radiotherapy has been widely accepted as the treatment modality for advanced NPC; however, treatment strategies for this disease have yet to be optimized [7-9].

The accurate prediction of prognosis and failure is crucial for optimizing therapy. In general, the 1997 AJCC staging system is the most widely used staging system for NPC [2]. However, the current TNM staging system is based on anatomic location and cranial nerve involvement that still has limitations. In addition to well established prognostic factors such as tumor stage, histopathologic type, and cranial nerve involvement, primary tumor volume has been recognized as a promising prognostic indicator in the treatment of NPC [10-13]. Since use of tumor volume could improve the ability of the current staging system to predict outcome, this study aimed to delineate the correlation between tumor volume, treatment outcome and chemotherapy cycles in NPC treated with multimodality therapy.

## Methods

### Patients

For this retrospective analysis, the treatment records of 142 patients with stage III-IV NPC (AJCC system) [2] from August 2000 to February 2007 in an institution were reviewed. Thirty-two patients were excluded because of distant metastasis present at initial diagnosis, loss to follow up, performance status >2, or a synchronous second primary tumor. The histological diagnosis of NPC was made by experienced pathologists. None of the patients received prior treatment for their cancer. All patients were informed about the treatment of neoadjuvant chemotherapy then concurrent chemoradiation (CCRT) and followed by adjuvant chemotherapy as being the definitive therapy for advanced disease, including the potential benefits and possible side effects. All patients were treated by multidisciplinary teams including a head and neck surgery team, radiation oncologists, medical oncologists and dieticians.

### Tumor volume measurement

All patients in this study underwent pre-treatment, contrast-enhanced CT scan that were done along the axial scan plain parallel to the infraorbital-meatal line extending from the skull base to the top of manubrium using 3-5 mm sections. Direct coronal scans also were taken to provide auxiliary information. One hundred millimeters of contrast medium were administrated with an injection rate between 1 mL per second and 2.5 mL per second after an initial 5-mL dose. Gross tumor volume of primary tumor plus retropharyngeal nodes (GTVprn) was included (depending on the imaging system) in the tumor volume measurement. First, manual tracing was performed using a graphic user interface, and area inside the outline was automatically labeled and calculated. The volume was calculated by multiplying the sum of all areas by the slice thickness (image reconstruction interval). All images were evaluated by two clinicians at least. A radiologist who specialized in head and neck cancer participated when the outline of tumor margin was unclear.

### Radiotherapy

An intensity-modulated radiation therapy (IMRT) technique and inverse planning system (PLATO, Nucleotron Inc, Veenendaal, Netherlands) were used for treatment delivery. The radiation field encompassed the primary tumor bed and neck lymph nodes. The prescribed dose of external beam treatment was 72 Gy to the gross tumor and positive neck nodes, 63 Gy to the clinical target volume, and 50-60 Gy to the clinically negative neck. Doses were delivered at 1.8 Gy/day for five consecutive days by a linear accelerator with patients lying supine with a mask. After 1-2 weeks of completing the external beam radiotherapy, an intracavitary brachytherapy boost (3.5 Gy to the submucosa 0.5 cm in 3 fractions) was prescribed if residual tumor was suspected. The intracavitary brachytherapy boost was conducted using high-dose-rate (HDR) afterloader unit (microSelectron-HDR, Nucleotron Inc, Veenendaal, Netherlands) containing an iridium-192 source.

### Chemotherapy

All consenting patients were eligible for chemotherapy if they met the following criteria: ECOG performance status ≤2, serum creatinine level <1.5 mg/dL, absolute neutrophil count ≥ 2000 cells/μL, and platelet count >10,000/μL. The chemotherapy protocol consisted of 6 monthly cycles of cisplatin (100 mg/m^2^/day) on Day 1 followed by 5-FU (1000 mg/m^2^/day) continuously infused for 5 consecutive days, in the presence of adequate hydration and anti-emetic drugs.

### Dose modification

Toxicity was evaluated using the common toxicity criteria of the National Cancer Institute. Both cisplatin and 5-FU were withheld if the absolute neutrophil count was <1500 cells/μL or if the platelet count was <75,000 cells/μL. Both agents were given at 70% of the initial dose if the neutrophil count was 1500-2000 cells/μL or if the platelet count was 75,000-100,000 cells/μL. Radiotherapy was withheld only if the neutrophil count was <1000 cells/μL or if the platelet count was <50,000 cells/μL. For grades 3 and 4 oropharyngeal mucositis or diarrhea, 5-FU was withheld until the symptoms improved. It was then restarted at 70% of the initial dose. For grades 3 and 4 renal toxicity, cisplatin was withheld until the creatinine was <1.5 mg/dL. It was administered at 70% of the initial dose thereafter.

### Patient evaluation

Survival was calculated from the date of diagnosis to the most recent follow-up or to the date of recurrence or death. The pattern of failure was defined according to the first site of failure: local failure defined as recurrence of the primary tumor or metastasis to regional lymph nodes; and distant failure indicating metastasis to any site beyond the primary tumor and regional lymph nodes. After recurrence or metastasis, patients were given salvage therapy as determined by their physicians.

### Statistical analysis

Different groups were compared with respect to base-line characteristics, with the *t*-test used for continuous variables and the chi-square test for categorical variables. The Kaplan-Meier method was used for survival analysis [14]. The difference between survival curves was determined using the log-rank test [15]. Multivariate analysis to identify significant prognostic factors was accomplished by Cox regression model. The receiver operating characteristic (ROC) curve analysis was applied to evaluate different cut-off point of tumor size in order to find the appropriate size for clinical application. SPSS 12.0 software (SPSS Inc, Chicago, IL, USA) was used for analysis of all data. Statistical significance was accepted as a *p *value of less than 0.05.

## Results

Patient baseline characteristics are presented in Table 1. Median patient follow-up at the commencement of the analysis was 38 months (range 4-107). All patients received the radiation. After extern beam radiotherapy, 13 of the 110 patients received an intracavitary brachytherapy boost (3.5 Gy to the submucosa 0.5 cm in 3 fractions) because residual tumor was suspected. Median cumulative radiation dose delivered over the study duration was 7200 cGy (range 6120-8250 cGy). For the rate of compliance with chemotherapy treatment, the patient received ≧ 4 and < 4 cycles of chemotherapy were 42% and 58%, respectively. During follow up, 7 patients had recurrence at the primary site, 23 patients had distant metastasis, and 5 patients combined recurrence with metastasis. The metastasis sites were the bone, lung, and liver. The 3-year overall survival, disease-free survival, local control, and distant metastasis-free rates in all patients were 67%, 66%, 80%, and 66%, respectively.

**Table 1:** Patient Characteristics

Patient characteristic	No. of patients (%)
All	110 (100)

Age	
Median	52
Range	26--73
Gender	
Male	71 (64.5)
Female	39 (35.5)
Histology	
WHO type I	3 (2.7)
WHO type II	84 (76.4)
WHO type III	23 (20.9)
Performance status (ECOG)	
0	103 (93.6)
1	4 (3.6)
2	3 (2.7)
AJCC 1997 T stage	
1	20 (18.2)
2	35 (31.8)
3	23 (20.9)
4	32 (29.1)
AJCC 1997 N stage	
0	2 (1.8)
1	3 (2.7)
2	75 (68.2)
3	30 (27.3)
AJCC 1997 Stage group	
III	61 (55.5)
IV	49 (44.5)

WHO, World Health Organization; ECOG, Eastern Cooperative Oncology Group; AJCC, American Joint Committee on Cancer.

**Figure 1:**
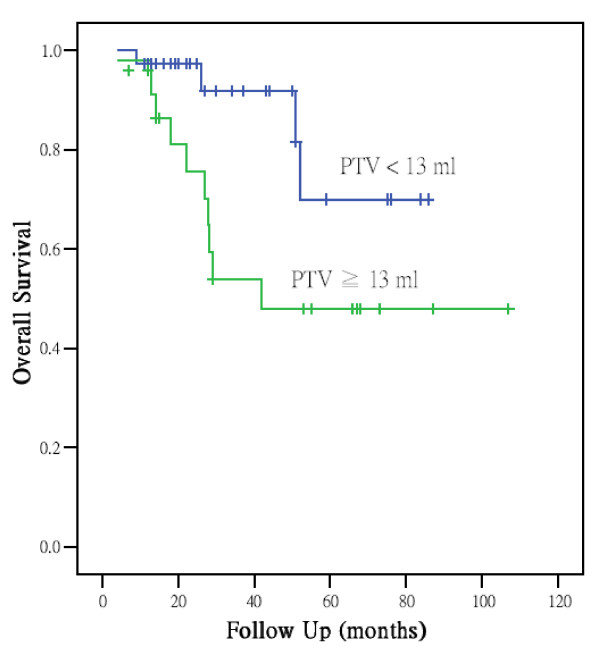
**Cumulative survival rates were stratified by primary tumor volume**. The 3-year overall survival in subgroups with GTVprn <13 ml and ≧ 13 ml were 92% and 54%. Large GTVprn (≧ 13 ml) was associated with a significantly poorer survival (p < 0.05).

**Figure 2:**
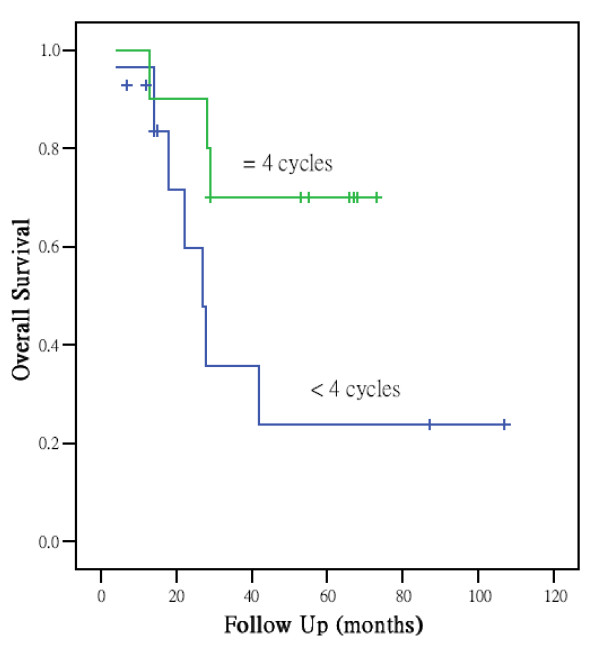
**Analysis of the subgroup with GTVprn ≧ 13 ml revealed better overall survival after ≧ 4 cycles of chemotherapy than after less than 4 cycles**. (p < 0.05).

Table 2 shows the T stage distribution and GTVprn for each stage. The median GTVprn of stage III to IV were 14.1 and 25.5 ml and corresponding ranges were 1.3-130.7 and 2.2-166.6 ml. The optimal GTVprn to find the appropriate size for clinical application was 13 ml. For GTVprn analysis, the 3-year overall survival, disease-free survival, local control, and distant metastasis-free rate in subgroups with GTVprn <13 ml and ≧ 13 ml were 92%/54%, 86%/25%, 95%/63%, and 88%/51%, respectively. Large GTVprn (≧13 ml) was associated with a significantly poorer local control, lower distant metastasis-free rate, and poorer survival (Figure 1). Besides, larger GTVprn (≧18 ml) was found to be correlation with N stage which was a significant prognostic factor on univariate analysis in distant metastasis free rate. Analysis of the subgroup with GTVprn ≧ 13 ml revealed better overall survival after ≧4 cycles of chemotherapy than after less than 4 cycles (Figure 2). Patients' 3-year overall survival, disease-free survival, local control, and distant metastasis-free rates were 70%/24%, 63%/30%, 78%/76%, and 65%/42%, respectively. A Cox proportional hazard regression model was constructed to calculate the relative risks and confidence intervals for different prognostic factors after controlling for age and gender. The results are summarized in Table 3. Only GTVprn was found to be an independent factor. Survival analysis demonstrated a significant difference in overall survival with larger tumor volume (risk ratio, 2.92; p = 0.02).

**Table 2:** T Stage and GTVprn

	GTVprn (ml)		Patients (%)	
	
T stage	Median	Range	GTVprn < 13 cm	GTVprn ≥ 13 cm
T1 (n = 20)	4.60	1.3--11.4	20 (100)	0 (0)
T2 (n = 35)	15.3	2.3--37.2	17 (49)	18 (51)
T3 (n = 23)	21.3	3.6--130.7	14 (61)	9 (39)
T4 (n = 32)	31.9	6.7--166.6	12 (38)	20 (62)

GTVprn, gross tumor volume of primary tumor plus retropharyngeal nodes

**Table 3:** Cox Proportional Hazard Model Analysis

Variables	Overall survival (RR, 95% CI)	*p*	Disease-specific survival (RR, 95% CI))	*p*	Disease-free survival (RR, 95% CI)	*p*	Loco-regional control (RR, 95% CI)	*p*	Distant metastasis-free survival (RR, 95% CI)	*p*
T1-2 vs. T3-4	1.29, 0.56-2.98	0.56	1.42, 0.41-4.86	0.58	0.85, 0.34-2.16	0.73	1.44, 0.27-7.64	0.67	1.00, 0.36-2.81	1.00
N0-2 vs. N3	2.47, 0.48-12.71	0.28	1.86, 0.15-23.26	0.63	2.85, 0.53-15.18	0.22	39.06, 1.55-983.27	0.03	2.77, 0.54-14.07	0.22
Cranial nerve involvement	0.71, 0.26-1.95	0.51	0.73, 0.17-3.13	0.67	0.65, 0.18-2.28	0.50	0.11, 0.006-2.10	0.14	0.95, 0.26-3.46	0.94
Supraclavicular nodes	0.74, 0.15-3.74	0.71	0.78, 0.06-9.59	0.85	0.45, 0.08-2.50	0.36	0.02, 0.001-0.87	0.04	0.68, 0.13-3.43	0.64
GTVprn (13 ml)	2.92, 1.22-6.98	0.02	4.10, 1.06-15.97	0.04	4.81, 1.73-13.36	< 0.01	16.83, 1.48-190.78	0.02	2.50, 0.84-7.43	0.10

RR, risk ratio; 95% CI, 95% confidence interval; GTVprn, gross tumor volume of primary tumor plus retropharyngeal nodes

## Discussion

The accurate prediction of prognosis and failure is crucial for optimizing therapy. We addressed the question of whether the AJCC staging system is adequate for predicting the prognosis of patients with NPC. NPC is often highly infiltrated and heterogeneous in all disease stages. Recently, tumor volume has been evaluated as a predictor because of the relationship of large volume with adverse biologic factors, including clonogen number, hypoxia, and radioresistance [11]. Several studies had demonstrated primary tumor volume could improve the current staging system [10-13]. Chua et al. found that primary tumor volume is an independent prognostic factor of local control and apparently more predictive than Ho's T stage classification [12]. Chen et al. also demonstrated that primary tumor volume predicted survival of patients with NPC with more accuracy than the AJCC staging system [10]. This indicates the limitation of the current TNM staging system based on anatomic location in separation of tumor bulk. Base on previous study, primary tumor volume was found to be a significant prognosis factor for treatment outcome and significantly correlated with T stage [13]. In the current study, additional testing was performed in an attempt to define the critical volume in the advanced NPC. We used a cut off value of 13 ml to divide patients into different prognostic groups. GTVprn ≧ 13 ml was associated with a significantly poorer local control, lower distant metastasis-free rate, and poorer survival. Since most of our patients had stage N2 and N3 tumors, we tried to determine whether GTVprn was correlated with N stage. However, GTVprn would be increased to 18 ml which could be correlated with N stage. Because the cut-off value of 13 ml could be used to predict and adjust treatment strategy. These results led us to hypothesize that GTVprn can refine the staging system and to speculate that micrometastases may sometimes occur before neck lymph node involvement is apparent.

Distant metastasis is an important concern that can influence survival. The reported frequency of distant metastases in patients with locally advanced NPC was greater than 30% with radiotherapy alone [7]. An autopsy series had shown a high rate of distant metastases (38-87%) involving virtually every organ [16]. Systemic chemotherapy was included in this investigation in an attempt to reduce the incidence of distant metastasis. In this study, failure pattern analysis revealed that the number of distant metastasis sites was greater than the number of local recurrence sites. The 3-year distant metastasis-free rate of 66% demonstrated that distant metastasis was still the main challenge in advanced NPC. In addition to delineate the cut off volume into different prognostic groups, we also analyzed the prognosis of subgroups with different conditions. We found that the subgroup with GTVprn ≧ 13 ml revealed longer survival after ≧ 4 cycles of chemotherapy than after less than 4 cycles. These results may hint the need for adequate systemic cycle regimes to eradicate micrometastases and improve survival. However, it is important to note that half of patients required treatment modification during chemotherapy or refused further treatment. More effective and safer drugs should be considered for integration into multi-modal treatment strategies.

Although incorporation of primary tumor volume could improve the accuracy of the current staging system, it still has some problems. One consideration is how tumor volume is quantitatively determined. Clinical care requires a classification system that reflects the current state of scientific knowledge and can guide clinical decision-making. NPC tumor volume measurement is a complicated procedure and the results may be affected by imaging modalities (CT or MR imaging), measuring protocols, and measurement techniques [17]. Rasch et al. indicated that MRI-derived tumor volume is smaller and has less interobserver variation than CT-derived tumor volume [18]. However, CT cannot be neglected because the geometric accuracy of the patient contour is poorer in MRI and lacks electron density information [10,19]. In addition, measurement of tumor volume is still time-consuming, labourintensive, and not widely available. Tumor volume definition and measuring protocols should be standardized in clinical practice. Lee et al. had tried to use simple measurement to evaluate of primary tumor volume and seemed feasible [13]. Otherwise, although large tumor volume was more commonly observed in the higher stages, there were large variation tumor volume and much overlapping among different stages [10-12]. Its means similar values of tumor volume could have different treatment response. Other factors such as tumor extension, intrinsic resistance, or hypoxia should be considered for integration into the staging system.

## Conclusions

Since this is a retrospective study, a number of factors in terms of patient and tumor characteristics could not be controlled and may have biased the results. Nevertheless, it appears that incorporation of tumor volume can further refine the staging system and adjust treatment strategy. For patients with large GTVprn (≧ 13 ml), the use of more effective and safer drugs with adequate systemic cycles is suggested.

## Competing interests

The authors declare that they have no competing interests.

## Authors' contributions

LCC and HSK developed the ideas for these experiments, performed much of the work, and drafted the manuscript. LMS, HSH, LHY and SYC designed the study, collected the data and interpreted the data. LCC performed the statistical analysis. All authors read and approved the final manuscript.
